# Asymmetric smooth pursuit eye movements and visual motion reaction time

**DOI:** 10.14814/phy2.14187

**Published:** 2019-07-28

**Authors:** Seiji Ono, Kenichiro Miura, Takashi Kawamura, Tomohiro Kizuka

**Affiliations:** ^1^ Faculty of Health and Sport Sciences University of Tsukuba Tsukuba Ibaraki Japan; ^2^ Department of Integrative Brain Science, Graduate School of Medicine Kyoto University Kyoto Japan; ^3^ Department of Pathology of Mental Diseases National Institute of Mental Health, National Center of Neurology and Psychiatry Tokyo Japan

**Keywords:** Motor control, perception, reaction time, smooth pursuit, visuomotor

## Abstract

Smooth pursuit eye movements often show directional asymmetry in pursuit initiation or steady‐state pursuit in both humans and monkeys. It has been demonstrated that the initial part of smooth pursuit is driven by visual motion related signals in cortical areas. Parietal cortex such as middle temporal (MT) and medial superior temporal (MST) areas are known to be involved in visual motion perception as well as pursuit initiation. Therefore, the purpose of this study is to determine whether directional asymmetry in pursuit initiation is associated with visual motion perception. We used a step‐ramp paradigm to induce horizontal smooth pursuit eye movements and then tested visual motion reaction time (RT). Visual motion RT was measured to the visual motion stimuli that moved leftward or rightward, which is an important parameter of our sensory motor processing based on visual motion perception. Nineteen healthy male subjects participated in the study. We found that some of our subjects showed directional asymmetries in initial pursuit acceleration between the leftward and rightward directions, which were consistent with an asymmetric bias in visual motion RT. Therefore, our results suggest that asymmetric pursuit initiation is associated with, at least in part, a bias of visual motion perception. These results could be due to a common neuronal pathway involved in both pursuit initiation and visual motion RT.

## Introduction

A smooth pursuit eye movement is induced when we look at a moving object to stabilize the image on or near the fovea. Pursuit initiation is supported by visuomotor systems where visual motion signals are transformed into pursuit commands (Krauzlis 2004; Lisberger 2010). The first 100 msec of pursuit tracking are defined as an open‐loop response that occurs before the time of a feedback signal. It is well‐known that the initial part of smooth pursuit is driven by visual motion related signals from cortical areas including the middle temporal (MT) and medial superior temporal (MST) areas (Newsome et al. 1985; Dursteler and Wurtz 1988). Those cortical visual motion‐related regions are also known to play a critical role in visual motion perception (Ding and Gold 2012; Liu and Pack 2017; Raghavan and Joshua 2017; Larcombe et al. 2018).

It has been demonstrated that smooth pursuit often shows directional asymmetry in initial acceleration or steady‐state velocity in both humans (Ke et al. 2013) and monkeys (Akao et al. 2007; Ono et al. 2012; Lee et al. 2013). Directional asymmetries are known to be more prominent for the vertical directions compared with the horizontal directions (Akao et al. 2007; Ke et al. 2013). For example, the overall initial pursuit response is biased toward the downward direction compared with the upward direction. In contrast, the overall horizontal pursuit shows a smaller bias between leftward and rightward directions. This is because the left/right bias may not be consistent among all subjects. In fact, one study has demonstrated that reading habits affect directional motion perception, which provides evidence of lateral motion bias (Morikawa and McBeath 1992). Furthermore, the speed of a moving target impacts pursuit characteristics and the degree of the asymmetry. Several other studies have also revealed directional anisotropies of motion responses in cortical areas and motion perception (Gros et al. 1998; Churchland et al. 2003; Dakin et al. 2010). Pursuit asymmetry is induced by several factors such as oculomotor deficits due to lesions of neurons in multiple brain sites in the smooth pursuit pathways. Furthermore, smooth pursuit adaptation, which is the directional specificity of adaptation to the demands of the visual environment, also leads to directional asymmetry. Previous studies have used a double‐step pursuit paradigm where the pursuit target begins moving at one speed for first 100 msec and then changes to either a higher or a lower speed (Fukushima et al. 1996; Kahlon and Lisberger 1996; Ono and Mustari 2012). This adaptation paradigm induces significant adaptive changes in pursuit initiation after 100–200 sequential trials. When this paradigm is applied for one direction (either leftward or rightward), the directional specificity of pursuit adaptation (asymmetric adaptation) is induced, while saccades are intact. However, it remains uncertain whether directional asymmetry in pursuit initiation is consistent with visual motion perception.

Visual‐motor reaction time (RT) has been utilized to evaluate speed of visuomotor processing which is measured as the time between the onset of the visual stimulus and the appearance of a motor response (Thorpe et al. 1996). Most studies that have dealt with visual stimulus used a light on/flash stimulus to evaluate motor reaction and visual perception. However, we are required to react to not only the contrast visual cue (e.g., light on RT task) but also visual motion. Reaction time to the visual motion stimulus is an important parameter of our sensory motor processing based on visual motion perception (Hulsdunker et al. 2017). In fact, visual motion sensitive cortical areas including MT, MST and the frontal eye field (FEF) are known to be involved in visual motion perception as well as pursuit initiation (Newsome et al. 1988; Churchland et al. 2003; Ono and Mustari 2009; Hedges et al. 2011). These cortical visual processing would influence motor reaction to visual motion stimulus. The current study attempted to determine whether directional asymmetries in pursuit initiation are associated with visual motion reaction time.

## Material and Methods

### Subjects

The subjects were nineteen male college students belonging to a baseball team with a mean age of 20.4 years (range 19–22) and they reported having normal or corrected to normal vision and no known motor deficits. The subjects were diagnosed neither as a stereoscopic problem nor strabismus. Of 19 subjects, 18 were right‐handed and one was left‐handed. They had no previous experience of our smooth pursuit and visual motion reaction time experiments and they were unaware of the specific aim of this study. All subjects gave their informed consent to participate in the experiment. This study was approved by the Research Ethics Committee at the Faculty of Health and Sport Science, University of Tsukuba.

### Smooth pursuit eye movement task

The subjects were seated 70 cm in front of a CRT monitor (22 inch, Diamond Pro 2070SB, Mitsubishi, refresh rate of 100 Hz, background mean luminance 60 cd/m^2^) with the head stabilized by a chin rest and a forehead restraint. Eye position signals from the right eye were calibrated by requiring the subjects to fixate a target spot (diameter of 0.3 deg) at known horizontal and vertical eccentricities in binocular viewing condition. The visual stimuli and target motion were generated by Psychophysics Toolbox extensions on MATLAB (Mathworks, MA). Smooth pursuit was produced by a step‐ramp paradigm (Rashbass 1961) with a constant speed of 18.5 deg/sec. The pursuit stimuli (diameter of 2 deg) were random dot fields (each dot, 5 × 5 pixels) whose contrast was modulated by a Gaussian window with a space constant of 20 pixels. Dots had a density of 50% and dot lifetime was equal to presentation duration. The subjects first fixated on a central stationary target (diameter of 0.3 deg) that appeared on uniform gray background for 1.0–1.5 sec and a pursuit target appeared 1.4 deg left or right from the center. The target then started to move either leftward or rightward. The subjects were instructed to track a moving target with their eyes. Ten trials were conducted for each direction. The leftward and rightward directions for smooth pursuit were randomized.

### Visual motion RT task

Visual motion RT was measured to the visual motion stimuli that moved leftward or rightward. The subjects were seated in front of a CRT monitor as a smooth pursuit task. They held buttons in each hand and were asked to press one of the buttons corresponding to the direction (leftward or rightward) of target motion. The subjects were asked to fixate on the stationary target and press the button as soon as possible once the target starts to move. A central fixation target appeared on uniform gray background for 1.0–1.5 sec and the target started to move leftward or rightward at a constant speed of 18.5 deg/sec. The moving target (diameter of 2 deg) was random dot fields whose contrast was modulated by a Gaussian window with a space constant of 0.5 deg. Ten successful trials were conducted for each direction and individual reaction time was determined by a mean value of each trial. The leftward and rightward directions were randomized.

### Light on RT task

Light on RT without visual motion was measured to the visual stimuli that appeared left or right side relative to the central fixation point. The light on RT task was a control task relative to the visual motion RT task. One hypothesis is that if the light on RT (without visual motion) is symmetric while the visual motion RT is asymmetric, this asymmetry could be due to the visual motion perception rather than hand dominance. The subjects were seated in front of a CRT monitor and held buttons in each hand and were asked to press one of the buttons corresponding to the location (left or right side) of a visual target. The central fixation target (diameter of 0.3 deg) appeared on uniform gray background for 1.0–1.5 sec and the second target (random dot fields, diameter of 2 deg) was presented at either the left or right side (2.8 deg eccentricity) relative to the central fixation point. The subjects were asked to fixate a stationary target and press the button as soon as possible once the second target appears. Ten successful trials were conducted for each side and individual reaction time was determined by a mean value of each trial. The left and right sides stimuli were randomized.

### Data collection and analysis

**Figure 1:**
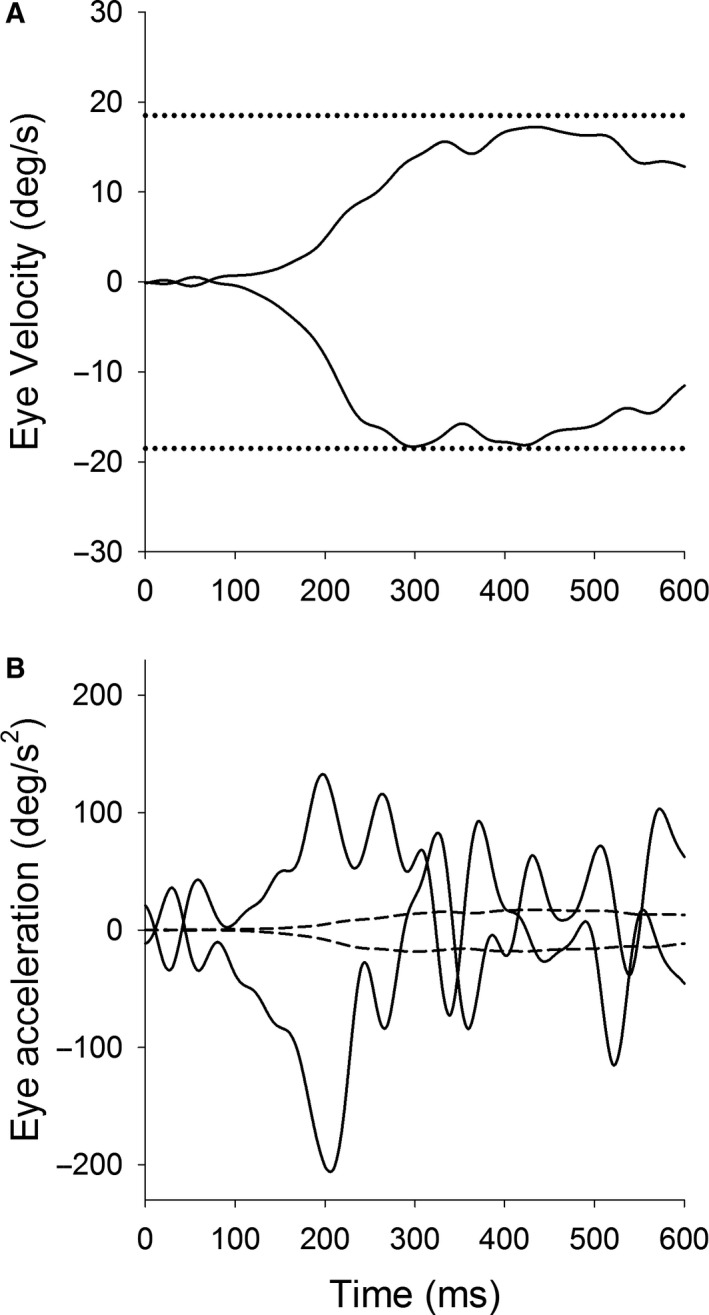
Smooth pursuit eye movements during a step‐ramp paradigm. Mean eye velocity traces are shown as a function of time in leftward and rightward directions (A). Target velocity (18.5 deg/sec) is indicated by the dotted lines. Mean eye acceleration traces are shown as a function of time in leftward and rightward directions (B). Solid and broken lines indicate eye acceleration and velocity traces, respectively. Upward deflections show rightward eye motion.

Eye movements were detected using a video based eye tracking system (see Matsuda et al. 2017). Eye position signals were digitized at 1 kHz with 16‐bit precision using CED‐Micro 1401 hardware (Cambrige Electronic Designs, Cambrige, England). Eye velocity and acceleration was generated by digital differentiation of the position arrays using a central difference algorithm in MATLAB (Mathworks, MA). Velocity and acceleration data were filtered using an 80‐point finite impulse response (FIR) digital filter with a passband of 80 Hz. Saccades were identified by velocity of 30 deg/sec or acceleration of 1000 deg/sec^2^, and were removed before averaging the data. Eye velocity traces were aligned on the onset of target motion and averaged from 10 trials with each direction (Fig. 1). We have used the convention of representing rightward eye position as positive values in our plots. Pursuit initiation (latency) during step‐ramp tracking was taken as the time that average eye speed reached > 3SD (3 times standard deviation) above the pretrial values during fixation. Initial acceleration was calculated as the average eye acceleration in the first 100‐msec period of pursuit. (Ono and Mustari 2007; 2012). Ten trials of rightward or leftward step‐ramp tracking were averaged to quantify initial acceleration and steady‐state velocity. Symmetry Index (SI) was calculated to demonstrate whether a directional bias of initial pursuit responses is consistent with that of visual motion RT, according to the following formula.

SI=(Lt-Rt)/(Lt+Rt)

where Lt and Rt are leftward and rightward pursuit responses/RTs respectively. A positive value of SI indicated a leftward bias, whereas a negative value of SI indicated a rightward bias. Similar indexes have been widely used to quantify ocular dominance (Sato and Stryker 2008) or gait symmetry (Nigg et al. 2013). Pearson’s correlation coefficient was used to examine the correlation between pursuit initiation and RT symmetry index. SigmaStat statistical software (Systat Software Inc, CA) was used for statistical analyses. All statistical tests were executed with an alpha level of 0.05.

## Results

### Smooth pursuit eye movement

This experiment focuses on the effect of visual motion direction (leftward and rightward) on smooth pursuit initiation. Figure 1 shows representative eye velocity and acceleration traces during step‐ramp tracking (ramp velocity = 18.5 deg/sec) to the leftward and rightward directions. Eye velocity and acceleration traces were aligned on the onset of target motion and averaged from 10 trials with each direction for one subject (Fig. 1). For example, steady‐state pursuit velocity (Fig. 1A) showed similar values for the rightward (16.8 ± 0.4 deg/sec) and the leftward (17.1 ± 0.8 deg/sec). In contrast, eye acceleration traces (Fig. 1B) showed that the leftward and rightward pursuit initiation (first 100 msec of tracking) were 121.7 ± 42.3 deg/sec^2^ and 73.2 ± 34.2 deg/sec^2^, respectively, indicating a directional asymmetry in pursuit initiation. Similarly, peak eye acceleration was also biased toward leftward motion (leftward, 207.5 deg/sec^2^; rightward, 133.4 deg/sec^2^).

**Figure 2:**
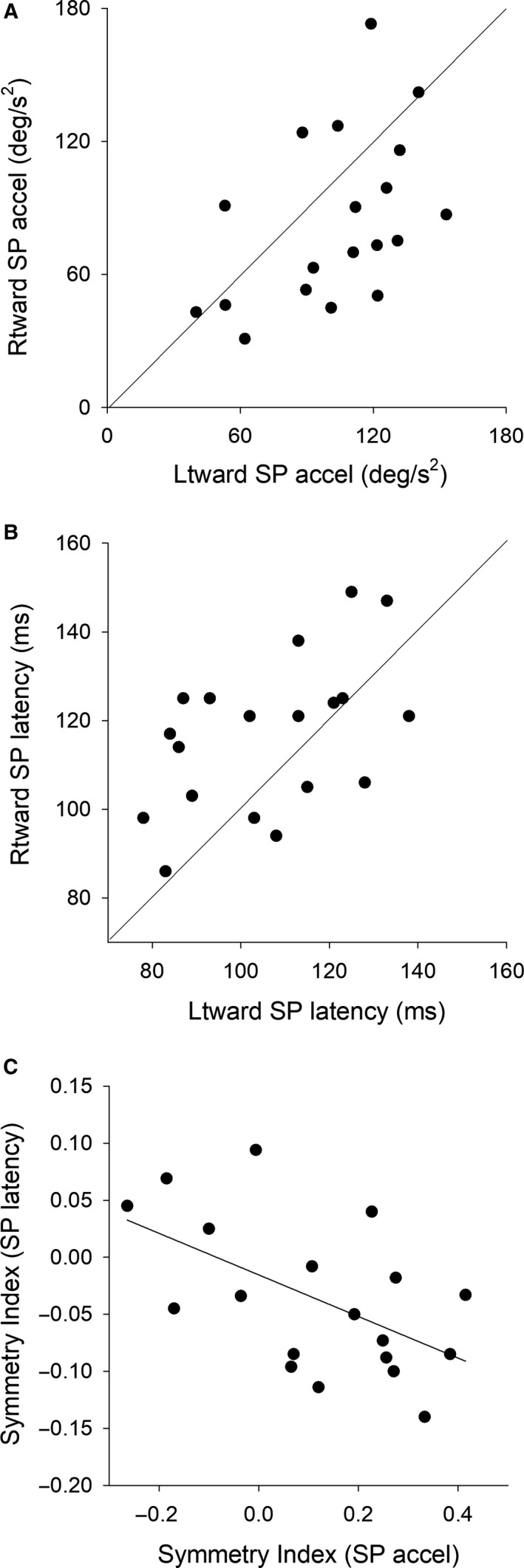
Mean values of initial acceleration (first 100 msec of tracking) (A) and pursuit latency (B) in each subject are plotted for leftward and rightward directions. The solid diagonal indicates the equality line. Symmetry Indexes (SI) of pursuit latency are plotted against SI of pursuit acceleration (C). The solid line indicates linear regression fit (*y* = −0.18*x* − 0.016). Pearson’s correlation analysis indicates a significant correlation between SI of pursuit acceleration and SI of pursuit latency [*r* (17) = −0.55, *P* < 0.05].

Scatter plots of the mean values of initial pursuit acceleration (first 100 msec of tracking) and pursuit latency in each subject are shown in Figure 2A and B respectively. Although pursuit initiation and latency seem to be affected by the motion direction, the data across the whole sample (*n* = 19) varied among the subjects. Therefore, we calculated Symmetry Index (SI) to demonstrate whether the directional bias of pursuit acceleration is consistent with that of latency. Figure 1C showed that there was a significant correlation between SI of pursuit acceleration and SI of pursuit latency (*r* = −0.55, *P* < 0.05, Pearson's correlation coefficient).

### Visual motion RT

**Figure 3:**
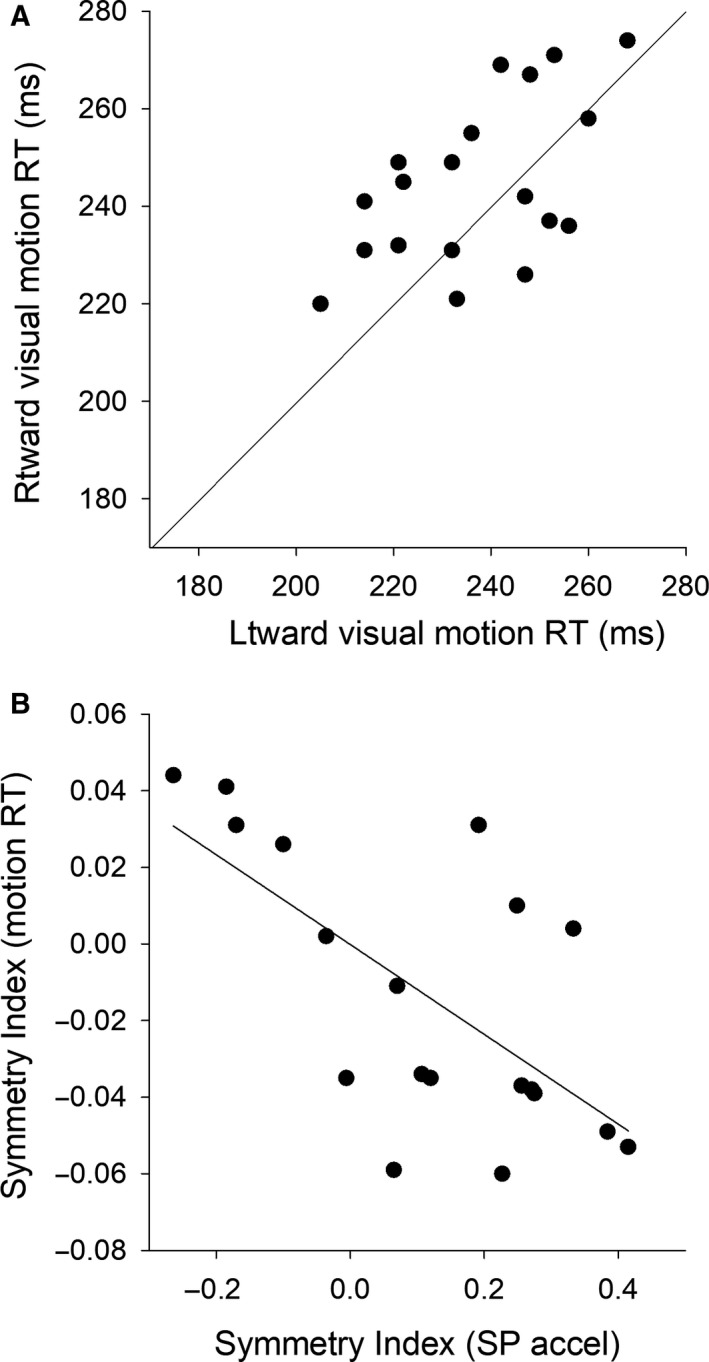
Visual motion RTs of 19 subjects, who were the same subjects in the smooth pursuit test, are plotted (A). The solid diagonal indicates the equality line. Symmetry Indexes (SI) of visual motion RT are plotted against SI of pursuit acceleration (B). The solid line indicates linear regression fit (*y* = −0.12*x* − 0.0002). Pearson’s correlation analysis indicates a significant correlation between SI of visual motion RT and SI of pursuit acceleration [*r* (17) = −0.65, *P* < 0.01].

Visual motion RTs of 19 subjects, who were the same subjects in the smooth pursuit test, were plotted in Figure 3A. In order to examine whether the directional bias of visual motion RT is consistent with that of pursuit acceleration, we calculated Symmetry Index (SI). Figure 3B shows individual data points of SI of visual motion RT plotted against SI of pursuit acceleration. Pearson’s correlation analysis showed that SI of visual motion RT was significantly correlated with SI of pursuit acceleration (*r* = −0.65, *P* < 0.01).

### Light on RT

**Figure 4:**
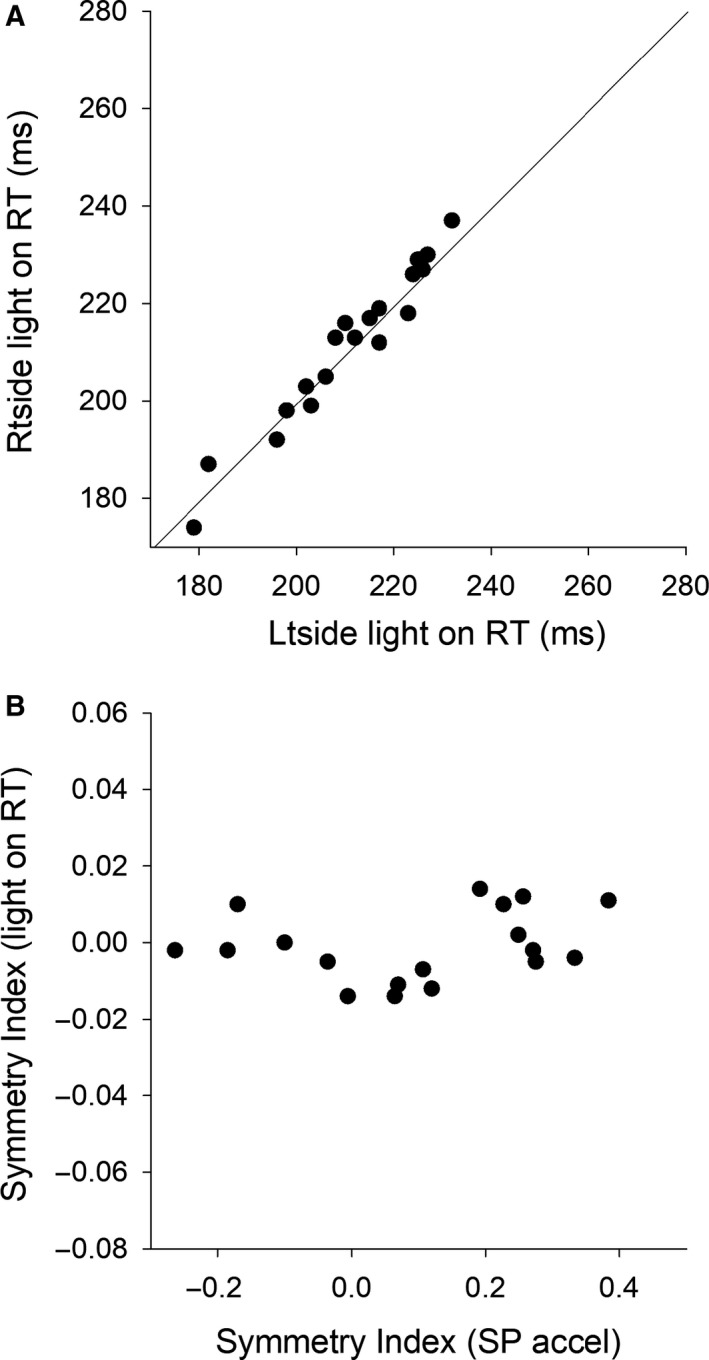
Light on RTs of 19 subjects, who were the same subjects in the smooth pursuit test, are plotted (A). The solid diagonal indicates the equality line. Symmetry Indexes (SI) of light on RT are plotted against SI of pursuit acceleration (B). Pearson’s correlation analysis indicates that there is no relationship between SI of light on RT and SI of pursuit acceleration [*r* (17) = 0.14, *P* = 0.57].

Light on RTs of 19 subjects, who were the same subjects in the smooth pursuit test, were plotted in Figure 4A. In contrast of visual motion RTs, most points fell near the equality line drawn on the plot, indicating that light on RTs did not show a bias between the left and right sides. Figure 4B shows individual data points of SI of light on RT plotted against SI of pursuit acceleration. Pearson’s correlation analysis showed that SI of light on RT was not correlated with SI of pursuit acceleration (*r* = 0.14, *P* = 0.57).

## Discussion

In this study, we attempted to determine whether asymmetric smooth pursuit initiation is associated with visual motion reaction time (RT). We used a step‐ramp paradigm with target velocity of 18.5 deg/sec to induce horizontal smooth pursuit eye movements. We then measured leftward and rightward visual motion RT.

### Possible mechanisms for asymmetric pursuit initiation

Previous studies have demonstrated that smooth pursuit often shows directional asymmetries for pursuit initiation and steady‐state pursuit in both humans and monkeys (Akao et al. 2007; Ono et al. 2012; Ke et al. 2013; Lee et al. 2013). This was the case even though the subjects have normal vision. Directional asymmetries are known to be more prominent for the vertical directions (up and downward) compared with the horizontal directions (left and rightward) (Ke et al. 2013). They suggest that pursuit asymmetries are due to adaptive responses to the requirements of the visual environments. Furthermore, the speed of a moving target impacts pursuit characteristics and the degree of the asymmetry.

Other studies have demonstrated that there are several factors to induce the horizontal pursuit asymmetry, which includes the deficit of the oculomotor neuronal circuit. For example, smooth pursuit is highly asymmetric in strabismic monkeys during monocular viewing conditions with much lower gain when the eyes moved in a temporal direction (Kiorpes and Movshon 2004; Hasany et al. 2008; Mustari and Ono 2011). Those studies suggest that such abnormal oculomotor behaviors could be due to differential loss of binocular neurons in multiple brain sites in the smooth pursuit pathways in cerebral cortex and cerebellum. Previous lesion studies have shown that inactivation of the cortical areas, pontine nuclei and the cerebellum produce characteristic ipsilesional deficits in pursuit initiation and steady‐state velocity (Suzuki et al. 1999; Takagi et al. 2000; Ono and Mustari 2007). The pontine nuclei are a critical site for relaying pursuit related signals from the medial superior temporal (MST) cortex to the floccular complex. Lesions anywhere along this circuit produce ipsilesional pursuit deficits, regardless of whether viewing is monocular or binocular.

Smooth pursuit adaptation (learning) is also an important factor to induce asymmetry of pursuit initiation. Previous studies have used a double‐step pursuit paradigm where the pursuit target begins moving at one speed for first 100 msec and then changes to either a higher or a lower speed (Fukushima et al. 1996; Kahlon and Lisberger 1996; Ono and Mustari 2012). This adaptation paradigm is designed to introduce additional retinal error motion, which induce significant adaptive changes in pursuit initiation after 100–200 sequential trials. When this paradigm is applied for one direction (either leftward or rightward), the directional specificity of pursuit adaptation (asymmetric adaptation) is induced. It has been suggested that the floccular complex and the oculomotor vermis in the cerebellum play an essential role in pursuit adaptation (Kahlon and Lisberger 2000; Takagi et al. 2000).

### Pursuit initiation and visual motion perception

Although the factors raised above are critical for the horizontal pursuit asymmetry, it remains uncertain whether the pursuit asymmetry is associated with the bias of visual motion perception. It has been demonstrated that reading habits affect directional motion perception, which provides evidence of lateral motion bias (Morikawa and McBeath 1992). Another study has also suggested that impaired visual motion perception may not affect smooth pursuit eye movements systems (Gonzalez et al. 2014). It has been known that initial part of smooth pursuit is dependent on retino‐striate projections that carry visual motion information to dorsal stream centers and oculomotor systems (Mustari et al. 1994; Krauzlis 2004). Visual motion information sensitive cortical areas including middle temporal (MT) and MST play crucial roles in pursuit initiation and motion perception (Dursteler and Wurtz 1988; Newsome et al. 1988; Churchland et al. 2003; Hedges et al. 2011). Cortical areas MT and MST have reciprocal connections with the frontal eye field (FEF) or frontal pursuit region, which plays an important role in visual motion perception (Ding and Gold 2012; Liu and Pack 2017; Raghavan and Joshua 2017; Larcombe et al. 2018). In fact, our previous study has reported that repeated pursuit trials lead to adaptive changes in the cortical visual response in the MST (Ono and Mustari 2016). It is most likely that a common neuronal pathway in cortical areas could be involved in both pursuit initiation and visual motion RT. Therefore, visual motion perception might provide a significant impact on pursuit initiation.

Our current study demonstrated that some of our subjects showed asymmetric smooth pursuit initiation between the leftward and rightward directions. Furthermore, Symmetry Index (SI) of pursuit acceleration was significantly correlated with SI of visual motion RT. In contrast, SI showed no significant correlation between pursuit acceleration and light on RT. Since the light on RT (without visual motion) was symmetric while the visual motion RT was asymmetric, this directional asymmetry in visual motion RT could be due to the bias of visual motion perception rather than hand dominance. Therefore, RT to the visual motion stimulus is an important indicator of our information processing speed and the appropriate motor response based on visual motion perception (Hulsdunker et al. 2017). Taken together, our results suggest that directional asymmetries in pursuit initiation are associated with, at least in part, a bias of visual motion perception.

## Conflict of Interest

None declared.
